# Assessment of Dental Arch Width in Individuals With Various Growth Patterns: A Cross-Sectional Study

**DOI:** 10.7759/cureus.96312

**Published:** 2025-11-07

**Authors:** Madhumitha Subramanian, Rajasekaran U B, Neetika Prabu, Arun Deepak, Vaibava Keerthana, Priyanka K, Ramkumar G

**Affiliations:** 1 Orthodontics and Dentofacial Orthopaedics, RVS Dental College and Hospital, Coimbatore, IND

**Keywords:** dental arch width, hyperdivergent, hypodivergent, normodivergent, orthodontic diagnosis, vertical growth pattern

## Abstract

Background: Dental arch width plays a vital role in orthodontic diagnosis, treatment planning, and post-treatment stability. Various skeletal growth patterns, hypodivergent, normodivergent, and hyperdivergent, are known to influence craniofacial morphology and dental arch dimensions. However, limited evidence exists comparing arch widths across these growth types, especially in South Indian populations.

Objectives: The objectives of this study are to assess maxillary and mandibular arch width measurements in normodivergent, hypodivergent, and hyperdivergent individuals and to compare variations in interpremolar, intermolar, premolar basal, and molar basal arch widths among these growth patterns.

Materials and methods: A retrospective cross-sectional study was conducted on 90 orthodontic patients (16-30 years) at RVS Dental College and Hospital using pretreatment casts and radiographs that were obtained from patient records for routine diagnostic and treatment purposes in the Department of Orthodontics and Dentofacial Orthopaedics. Pretreatment dental casts and corresponding lateral cephalograms were screened to identify cases fulfilling the required diagnostic and record quality standards. All data were anonymized prior to evaluation, and no direct patient contact or intervention was involved in the study. Based on cephalometric parameters (FMA, SN-GoGn, Y-axis), subjects were divided into three groups (n=30 each): normodivergent, hypodivergent, and hyperdivergent. Dental arch widths were measured on casts, including interpremolar, intermolar, premolar basal, and molar basal widths in both arches. Statistical analysis was done using IBM SPSS Statistics for Windows, Version 27 (Released 2020; IBM Corp., Armonk, New York, United States). One-way ANOVA with post hoc Tukey tests was applied, and p<0.05 was considered significant.

Results: Hypodivergent subjects consistently exhibited the greatest arch widths, while hyperdivergent subjects showed the narrowest. In the maxilla, the mean interpremolar width was 43.31±0.40 mm (hypodivergent), 42.81±0.39 mm (normodivergent), and 40.94±0.49 mm (hyperdivergent) (p<0.01). A similar trend was observed for intermolar, premolar basal, and molar basal widths. Mandibular arch widths followed the same pattern, with hypodivergent > normodivergent > hyperdivergent (p<0.01 for all parameters). Post hoc tests confirmed statistically significant differences across groups.

Conclusion: Dental arch widths vary significantly with various skeletal growth patterns. Hypodivergent individuals possess the widest arches, hyperdivergent the narrowest, and normodivergent intermediate dimensions. These findings emphasize considering vertical skeletal growth in orthodontic diagnosis, archwire selection, and long-term treatment stability.

## Introduction

Orthodontics is concerned with the diagnosis and correction of malocclusion to enhance dental function, esthetics, and stability of outcomes. Dental arch sizes and patient facial form are among the most important areas of orthodontic diagnosis. Dental arch width and facial form are also essential determinants of whether treatment will be successful and stable in the long term [1].

Arch form describes the three-dimensional position and interrelationship of teeth in the dental arches. Its width is a critical determinant of occlusal harmony, smile esthetics, and space available for alignment of teeth. Changes in dental arch width, as a result of growth, skeletal relationships, or treatment mechanics, can lead to spacing, crowding, crossbites, or posttreatment relapse. Therefore, a clear comprehension of the forces that affect arch width is imperative in orthodontic planning [2].

According to Hawley [3], the ideal arch width could be described as an equilateral triangle, with its base representing the intercondylar width. The lower anterior teeth were positioned along an arc of a circle, with the radius determined by the combined widths of the lower incisors and canines, while the premolars and molars were aligned progressively toward the center. This geometric method emphasizes the importance of the arch width in creating harmony between the dentition and craniofacial structures.

Facial morphology has long been accepted to be the product of an individual's genotype and its phenotypic expression. It is also generally accepted that there is a correlation between the functional capacity and the size of the masticatory muscles with craniofacial morphology. On the basis of vertical proportions, three fundamental facial morphologies are known as short, average, and long face types.

Long face type is defined by hypervertical growth, anterior open bite, elevated sella-nasion-mandibular plane (SN-MP) angle, elevated gonial angle, and a high maxillary/mandibular plane angle. Short face type is marked by diminished vertical growth, deep overbite, low facial height, and a low SN-MP angle. Average face type falls in between, which is indicative of balanced growth and harmonious proportions.

Growth patterns are also traditionally categorized as horizontal, vertical, and average types, depending on cephalometric values like Y-axis, mandibular plane angle (FMA, SN-GoGn), and facial height proportion [4].

Numerous studies have emphasized the association of skeletal growth patterns with arch form [5]. Hypodivergent patients have been found to have wider dental arches with stronger musculature and horizontal rotation of the mandible. Hyperdivergent patients will have narrower arches, especially of the posterior segments, due to less musculature and vertical mandibular growth. Normodivergent patients are intermediate between these two extremes, with symmetrical arch width [1]. These differences are important in that orthodontic intervention, which changes arch width without regard to the underlying growth pattern, can cause instability and relapse [6].

The significance of arch width in orthodontics is great. Respecting or preserving the patient's native arch form is paramount to long-term stability of outcome. Arch expansion may be more stable in hypodivergent patients because of better support from the skeletal and muscular components, or in the hyperdivergent patient, where expansion tends to be less stable and relapse tends to occur. Although this study did not assess post-treatment stability directly, the findings have implications for clinical decision-making. The observed variation in arch width with growth pattern suggests that individualized archwire selection may enhance long-term stability, as reported in previous literature [6]. Thus, knowing how arch width varies with different types of skeletal growth can assist clinicians in making the right choice of archwire form, determining when expansion is possible, and reducing post-treatment relapse [7].

The majority of orthodontic investigations have previously centered on sagittal skeletal relationships, i.e., Class I, II, or III malocclusions. The vertical dimension, however, though equally relevant, has received relatively less attention. Skeletal growth patterns tend to present in the form of open bites, deep bites, or long-face syndromes, affecting not just esthetics and function, but also underlying dental arch morphology. An exploration of the interaction between growth pattern and arch width can offer useful insights for customized orthodontic treatment planning [8].

Against this backdrop, the current study will measure and compare dental arch widths between individuals with varying skeletal growth patterns: normodivergent, hypodivergent, and hyperdivergent.

Null hypothesis

There is no significant difference in the dental arch width among individuals with various skeletal growth patterns.

The hypotheses were two-sided, testing for any directional difference among growth patterns, consistent with the exploratory nature of the study.

## Materials and methods

This retrospective cross-sectional study was conducted in the Department of Orthodontics and Dentofacial Orthopaedics, RVS Dental College and Hospital. The study was carried out over a period of three months, commencing on April 2, 2025, and concluding on June 27, 2025. The study sample was retrospectively selected from the departmental archives of orthodontic patient records at the Department of Orthodontics and Dentofacial Orthopaedics, RVS Dental College and Hospital. Pretreatment dental casts and corresponding lateral cephalograms were screened to identify cases fulfilling the required diagnostic and record quality standards. A total of 90 samples were included, and patients were divided into three groups of 30 each, based on vertical growth patterns assessed from cephalometric analysis. An a priori power analysis (G*Power 3.1) has been added. It showed that 27 participants per group were required to detect a 1.5 mm difference (SD = 1.8 mm, α = 0.05, power = 80%). Our sample of 30 per group met this requirement. Ethical approval for this research was obtained from the Institutional Review Board of RVS Dental College and Hospital (Ref No: IRB22S/111). All data were anonymized prior to evaluation, and no direct patient contact or intervention was involved in the study.

Inclusion and exclusion criteria

The study included patients exhibiting a complete permanent dentition, excluding the third molars. Only cases with good-quality pretreatment dental casts and lateral cephalograms were considered for analysis. The age of the participants ranged from 16 to 30 years.

Crowding was assessed through cast analysis using arch length discrepancy, calculated as the difference between the tooth width sum and available arch length. Only cases within the range of ≤9 mm were included. Patients presenting with anterior or posterior crossbites, excessive crowding or spacing greater than 9 mm, or any craniofacial anomalies or syndromes were excluded from the study. Individuals with a history of trauma to the dentofacial region or deleterious oral habits resulting in cuspal wear were also excluded. Dental casts with worn or altered cusps were excluded to ensure accuracy of cusp-based width measurements. Subjects with missing or restored first premolars/molars were excluded; no substitution with second premolars or other teeth was performed. Furthermore, dental casts showing gross caries, extensive restorations, or the presence of prosthetic crowns in the posterior teeth were not included in the sample.

Grouping of samples

Samples were divided into three groups of 30 each. Group 1: Average growth pattern (Normodivergent): FMA =25°, SN-GoGn =32°, Y-axis = 53°; Group 2 - Horizontal (Hypodivergent) growth pattern: FMA < 25°, SN-GoGn < 32°, Y-axis < 53°; Group 3 - Vertical (Hyperdivergent) growth pattern: FMA > 25°, SN-GoGn > 32°, Y-axis > 53°.

Cases were categorized based on overall cephalometric consistency using FMA, SN-GoGn, and Y-axis. When a slight variation occurred among these parameters, classification was made considering the predominant or average trend of the three values. Borderline measurements at the cutoff were regarded as normodivergent, aligning with conventional cephalometric grouping approaches.

Frankfort mandibular plane angle (FMA)

The FMA is the angle between the Frankfort horizontal plane (FH) and the mandibular plane.

Frankfort horizontal plane

The Frankfort horizontal plane is the line joining from external auditory meatus to the orbitale.

Mandibular plane

The mandibular plane is the line passing tangent to the lower border of the mandible.

Sella-Nasion to Gonion-Gnathion angle (SN.GoGn)

SN.GoGn is the angle between SN and Steiner's mandibular planes (GoGn).

Y-axis

Y-axis is the angle between the S-Gn and SN planes.

Reference points and measurements

**Figure 1:**
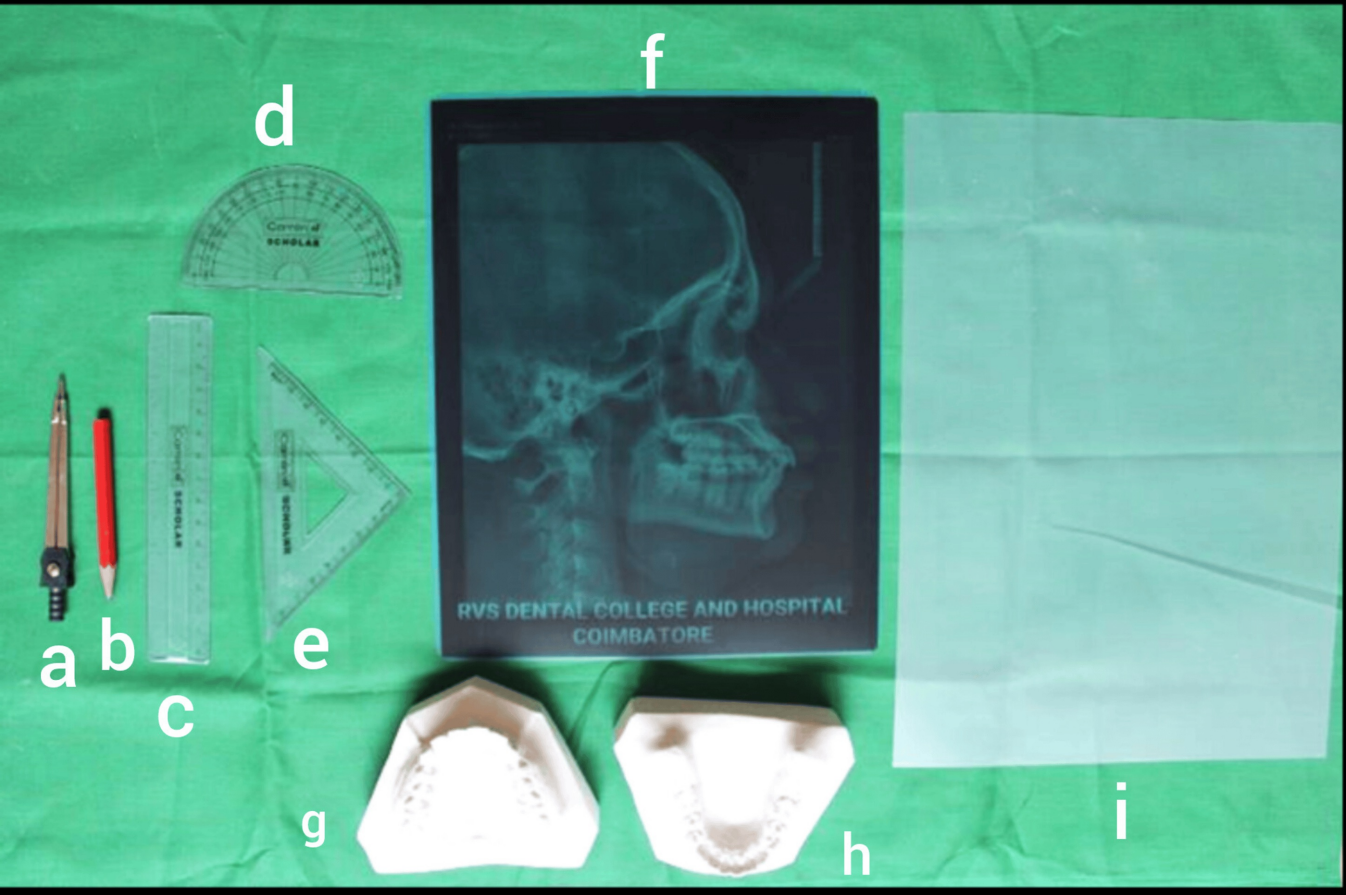
Armamentarium used for measurements a) Divider, b) 3H drawing pencil, c) Scale, d) Protractor, e) Set square, f) Lateral cephalogram, g) Maxillary study model, h) Mandibular study model, and i) Lead acetate tracing sheet

All reference points were marked on the study casts with a sharp 3H pencil. Measurements were carried out using standard orthodontic instruments such as a ruler, protractor, set square, compass, and acetate tracing sheets (Figure 1). This ensured precise linear and angular measurements on both dental casts and cephalometric tracings. The divider points were verified against the scale before each session to maintain accuracy. To reduce inter-observer variability, all measurements were performed by the same operator. Repeated measurements on 10 randomly selected casts showed ICC values above 0.90 and Dahlberg error between 0.15 and 0.25 mm, indicating excellent repeatability.

**Figure 2:**
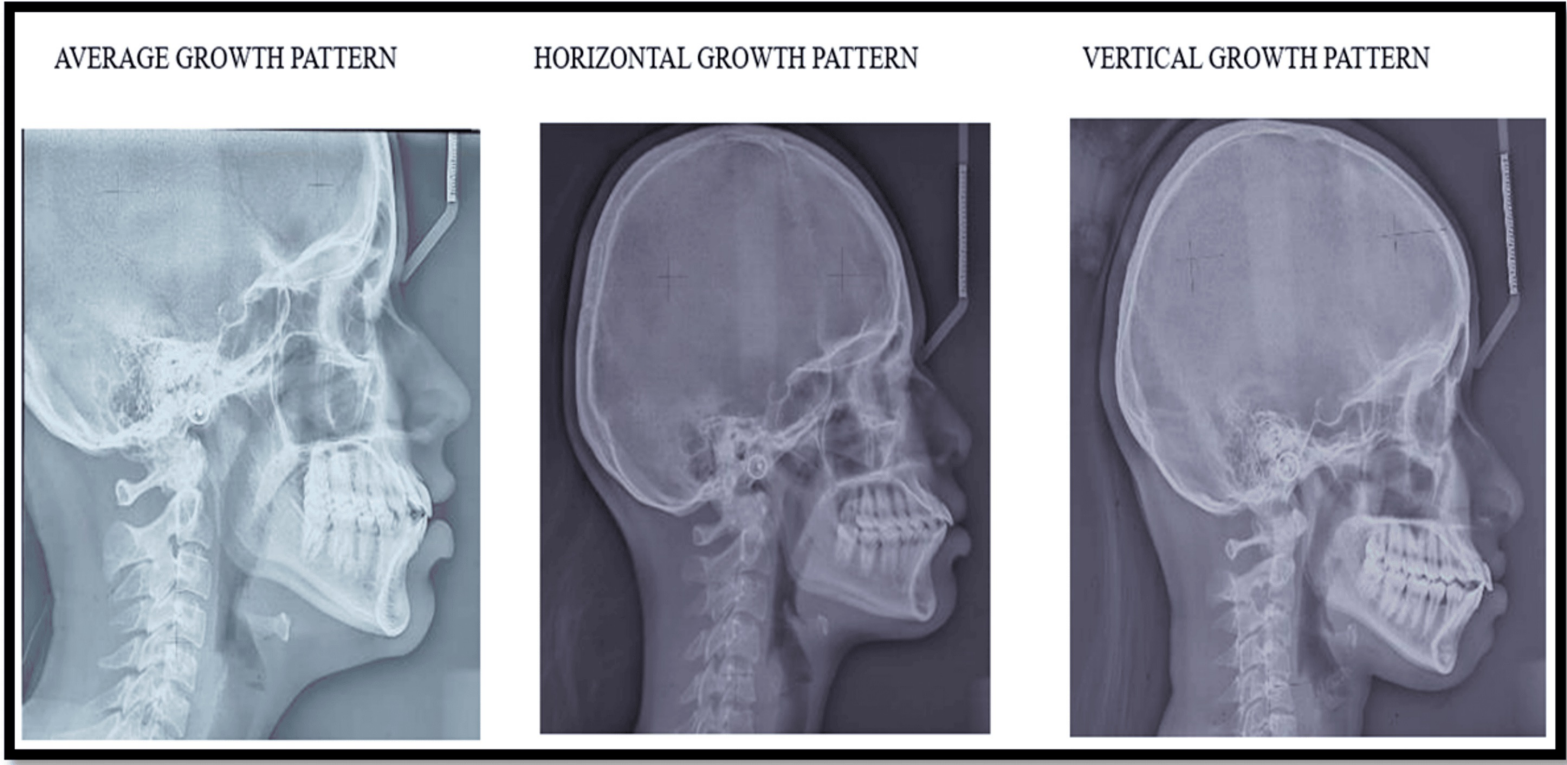
Lateral cephalograms showing three growth patterns

Cephalometric analysis was performed using lateral cephalograms (Figure 2). Reference landmarks were traced manually on acetate tracing sheets placed over the radiographs, and growth patterns were confirmed by measuring FMA, SN-GoGn, and Y-axis angles with the help of protractors and set squares. All cephalometric tracings were carried out by the same examiner to ensure consistency. While blinding was not formally implemented, the examiner followed a standardized measurement protocol for all radiographs. Repeated tracing of randomly selected samples demonstrated excellent intra-examiner consistency (ICC > 0.90), confirming reliability.

**Figure 3:**
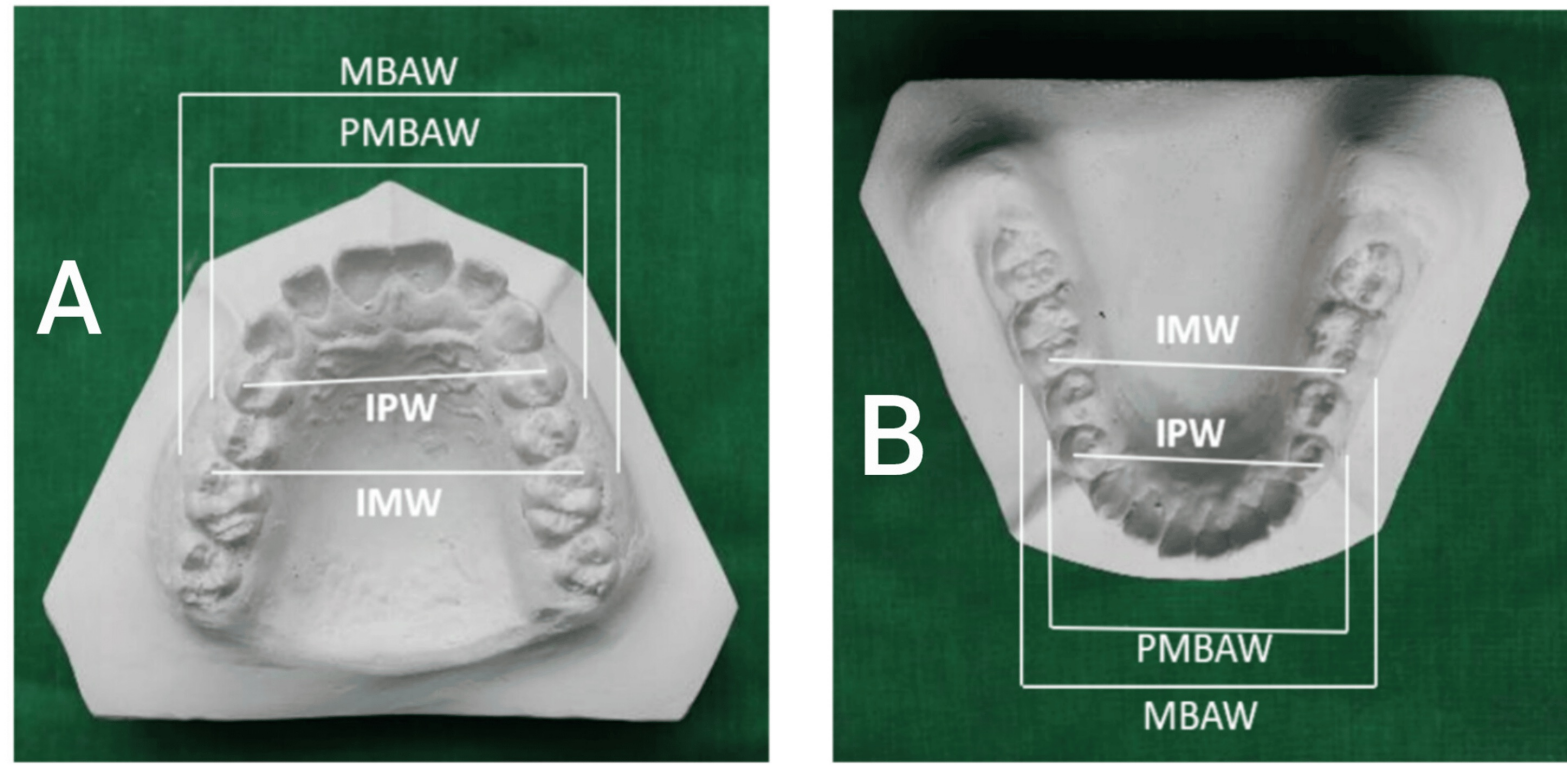
Arch width measurements in maxillary and mandibular casts A: Maxillary cast. B: Mandibular casts IPW: Inter-premolar arch width; IMW: Inter-molar arch width; PMBAW: Premolar basal arch width; MBAW: Molar basal arch width

The following arch width dimensions were measured for both maxillary and mandibular dental casts (Figure 3).

Inter-premolar arch width (IPW)

It is the distance between the buccal cusp tips of the right and left first premolars.

Inter-molar arch width (IMW)

It is the distance between the mesiobuccal cusp tips of the right and left first molars.

Premolar basal arch width (PMBAW)

It is the distance between two points at the mucogingival junctions, located above the interdental contact points of the maxillary first and second premolars and below the interdental contact points of the mandibular first and second premolars.

Molar basal arch width (MBAW)

It is the distance between two points at the mucogingival junctions, located above the mesiobuccal cusp tips of the maxillary first molars and below the mesiobuccal cusp tips of the mandibular first molars.

Basal arch width points were defined at the mucogingival junction directly above (maxilla) or below (mandible) the mesiobuccal cusp tips of the respective teeth, following the method of Prasad et al. (2013) [1]. This definition minimizes soft-tissue variation on study casts. A schematic representation of basal landmarks is provided in Figure 3.

Statistical analysis

Data obtained were tabulated and subjected to statistical analysis using IBM SPSS Statistics for Windows, Version 27 (Released 2020; IBM Corp., Armonk, New York, United States). Descriptive statistics (mean and standard deviation) were calculated for each parameter. Normality of variances was verified using the Shapiro-Wilk test. Inter-group comparisons were carried out using one-way ANOVA, followed by the post hoc Tukey test for multiple comparisons. In addition to ANOVA and Tukey post hoc tests, effect sizes and 95% confidence intervals were calculated. A p-value of <0.05 was considered statistically significant.

## Results

A total of 90 subjects (30 normodivergent, 30 hypodivergent, 30 hyperdivergent) were analyzed for interpremolar, intermolar, premolar basal, and molar basal arch widths.

Normality of the data was assessed using the Shapiro-Wilk test. As all variables showed p-values greater than 0.05, the data were considered normally distributed. Therefore, descriptive statistics are presented as mean ± standard deviation, and inferential statistics were performed using parametric tests (one-way ANOVA with Tukey post hoc analysis).

Maxillary arch widths

In the maxilla, hypodivergent subjects consistently exhibited the highest arch width values, while hyperdivergent subjects showed the lowest. The mean interpremolar dental arch width was 43.31 ± 0.40 mm in hypodivergent, 42.81 ± 0.39 mm in normodivergent, and 40.94 ± 0.49 mm in hyperdivergent individuals.

Similarly, the intermolar dental arch width was greatest in hypodivergent subjects (53.02 ± 0.60 mm), followed by normodivergent (52.25 ± 0.51 mm), and least in hyperdivergent subjects (50.80 ± 0.43 mm). The premolar basal arch width was widest among hypodivergent individuals (51.47 ± 0.55 mm), compared with 48.42 ± 0.84 mm in normodivergent and 46.45 ± 0.75 mm in hyperdivergent subjects. It is summarized in Table 1. One-way ANOVA showed significant differences among growth patterns for all maxillary arch width parameters (p < 0.01).

**Table 1:** Maxillary arch width measurements by growth pattern mm: millimeter Intergroup comparison by one-way ANOVA

Arch Width Parameters	Normodivergent Mean± SD	Hypodivergent Mean± SD	Hyperdivergent Mean± SD	p-value (One-way ANOVA)	F value
Interpremolar Dental Arch Width (mm)	42.81± 0.39	43.31 ± 0.40	40.94 ± 0.49	<0.01	251.964
Intermolar Dental Arch Width (mm)	52.25 ± 0.51	53.02 ± 0.60	50.80 ± 0.43	<0.01	139.239
Premolar Basal Arch Width (mm)	48.42 ± 0.84	51.47± 0.55	46.45± 0.75	<0.01	364.469
Molar Basal Arch Width (mm)	58.59 ± 0.76	58.65 ± 0.59	57.81 ± 0.50	<0.01	16.701

Post hoc Tukey tests revealed that hypodivergent subjects had significantly greater values compared with normodivergent and hyperdivergent groups (p < 0.01), while hyperdivergent individuals consistently demonstrated the lowest measurements across all parameters. The p-value is given in Table 2.

**Table 2:** Post hoc Tukey tests for multiple comparisons of maxillary arch width measurements mm: millimeter ** highly significant

Arch Widths (in mm)	Growth Pattern (i)	Growth Pattern (j)	Mean Difference(i-j)	p-Value
Interpremolar Dental Arch Width (mm)	Normodivergent	Hypodivergent	-.50000	<0.01
		Hyperdivergent	1.87000	<0.01
	Hypodivergent	Hyperdivergent	2.37000	<0.01
Intermolar Dental Arch Width (mm)	Normodivergent	Hypodivergent	-.77333	<0.01
		Hyperdivergent	1.44333	<0.01
	Hypodivergent	Hyperdivergent	2.21667	<0.01
Premolar Basal Arch Width (mm)	Normodivergent	Hypodivergent	-3.05000	<0.01
		Hyperdivergent	1.97333	<0.01
	Hypodivergent	Hyperdivergent	5.02333	<0.01
Molar Basal Arch Width (mm)	Normodivergent	Hypodivergent	-.06000	<0.01
		Hyperdivergent	.78333	.000**
	Hypodivergent	Hyperdivergent	.84333	.000**

A similar trend was observed in the mandible. The mean interpremolar dental arch width was 36.25 ± 0.48 mm in hypodivergent subjects, 35.60 ± 0.51 mm in normodivergent, and 34.54 ± 0.38 mm in hyperdivergent individuals. Intermolar dental arch width was highest in the hypodivergent group (48.62 ± 0.53 mm), followed by normodivergent (47.77 ± 0.72 mm) and lowest in hyperdivergent (46.90 ± 0.50 mm).

The premolar basal arch width measured 39.68 ± 0.51 mm in hypodivergent, 38.79 ± 0.58 mm in normodivergent, and 37.80 ± 0.47 mm in hyperdivergent subjects. The molar basal arch width was again widest in hypodivergent individuals (53.19 ± 0.72 mm), intermediate in normodivergent (52.18 ± 0.78 mm), and narrowest in hyperdivergent (50.84 ± 0.68 mm). It is given in Table 3. One-way ANOVA showed significant differences among growth patterns for all mandibular arch width parameters (p < 0.01).

**Table 3:** Mandibular arch width measurements by growth pattern mm: millimeter Intergroup comparison: One-way ANOVA

Arch Width Parameters	Normodivergent Mean± SD	Hypodivergent Mean± SD	Hyperdivergent Mean± SD	p-value (One-way ANOVA)	F value
Interpremolar Dental Arch Width (mm)	35.60 ± 0.51	36.25 ± 0.48	34.54 ± 0.38	<0.01	104.177
Intermolar Dental Arch Width (mm)	47.77 ± 0.72	48.62 ± 0.53	46.90 ± 0.50	<0.01	62.708
Premolar Basal Arch Width (mm)	38.79 ± 0.58	39.68 ± 0.51	37.80 ± 0.47	<0.01	97.188
Molar Basal Arch Width (mm)	52.18 ± 0.78	53.19 ± 0.72	50.84 ± 0.68	<0.01	78.957

Post hoc Tukey tests revealed that hypodivergent subjects had significantly greater values compared with normodivergent and hyperdivergent groups (p < 0.01), while hyperdivergent individuals consistently demonstrated the lowest measurements across all parameters. The p-value is given in Table 4.

**Table 4:** Post hoc Tukey tests for multiple comparisons of mandibular arch width measurements for three growth patterns mm-millimeter

Arch Widths	Growth Pattern (i)	Growth Pattern (j)	Mean Difference(i-j)	p-Value
Interpremolar Dental Arch Width (mm)	Normodivergent	Hypodivergent	-.65000	<0.01
		Hyperdivergent	1.05667	<0.01
	Hypodivergent	Hyperdivergent	1.70667	<0.01
Intermolar Dental Arch Width(mm)	Normodivergent	Hypodivergent	-.85333	<0.01
		Hyperdivergent	.86333	<0.01
	Hypodivergent	Hyperdivergent	1.71667	<0.01
Premolar Basal Arch Width (mm)	Normodivergent	Hypodivergent	-.89667	<0.01
		Hyperdivergent	.98333	<0.01
	Hypodivergent	Hyperdivergent	1.88000	<0.01
Molar Basal Arch Width (mm)	Normodivergent	Hypodivergent	-1.01000	<0.01
		Hyperdivergent	1.34000	<0.01
	Hypodivergent	Hyperdivergent	2.35000	<0.01

These findings indicate that both maxillary and mandibular dental arch widths vary significantly across different skeletal growth patterns. Hypodivergent subjects consistently demonstrated the greatest arch dimensions, followed by normodivergent subjects, while hyperdivergent individuals had the narrowest arches. Each pairwise comparison revealed statistically significant differences (p < 0.01).

## Discussion

The current cross-sectional assessment compared maxillary and mandibular dental arch width in patients with three growth patterns-hypodivergent, normodivergent, and hyperdivergent. The results showed that hypodivergent patients had the maximum interpremolar, intermolar, premolar basal, and molar basal arch widths throughout, while the dimensions were minimum in hyperdivergent patients. Normodivergent patients had intermediate values. These variations were found to be statistically significant in both maxillary and mandibular dimensions, thus establishing a robust relationship between dental arch morphology and facial growth pattern.

Khan et al. performed a cross-sectional analysis in 110 patients to evaluate arch width differences among various skeletal vertical patterns by using the SNMP angle for the assessment of the vertical plane [9]. Their findings revealed that while men had larger intermolar arch widths than women, the entire difference in arch width among different vertical growth patterns was not statistically significant. Strikingly, their data showed that patients with normally angled SNMP had larger arch widths, while those with low and high SNMP angles had relatively lower arch sizes.

In comparison with the current study, some similarities and differences are noted. Consistent with the findings of Khan et al., our data also indicate relatively greater arch sizes among normodivergent (normal growth) subjects. In contrast to their indication of negligible differences between groups, our investigation found more pronounced differences, especially when hypodivergent and hyperdivergent subjects were compared. This difference could be due to a difference in sample size, age distribution, and method used. For example, whereas Khan et al. used only the SNMP angle in exclusivity for categorization, the present study employed various cephalometric parameters such as Y-axis, FMA, and SN-GoGn, thus potentially providing a more inclusive measure of vertical skeletal patterns.

Recently, Taj et al. (2024, Peshawar) compared arch width differences in 90 untreated adults categorized into normodivergent, hypodivergent, and hyperdivergent types using the FMA [10]. They found statistically significant variations in maxillary inter-canine, maxillary intermolar, and mandibular intermolar widths, the latter two being narrower for hyperdivergent subjects. A significant difference was not found for mandibular intercanine width. Their findings support this study closely, notably in verifying the trend for high-angle cases to have narrower arch shapes. In combination, these findings support the belief that posterior arch size is under the significant influence of vertical skeletal pattern.

Additionally, Aggarwal et al. (2019) [11] carried out a study among the Solan population to evaluate dental and alveolar arch width among 45 patients with various vertical growth patterns utilizing pretreatment casts and cephalograms. They established that hypodivergent patients had the largest dental and alveolar arch widths, while hyperdivergent patients had the smallest. Yet, the intergroup variation was statistically not significant. Contrarily, in the present study, there were significant differences in all parameters. This discrepancy can be attributed to lesser sample size and a difference in the region in Solan's study, while our greater sample size and wider cephalometric criteria gave a stronger correlation.

Our findings conform to previous studies carried out on various populations. Nasby et al. described larger circumferences of the maxillary and mandibular arches in individuals with a low mandibular plane angle, indicating wider arches in hypodivergent individuals [12]. Likewise, Christie identified wider maxillary and mandibular widths in brachyfacial (horizontal growers) males compared to dolichofacial (vertical growers) men, which supports the present findings [13]. The current research also echoes a South Indian study [1], where hypodivergents always had wider arch forms than hyperdivergents, pointing towards the importance of musculature and rotation of the mandible in arch width development.

Furthermore, Ocak et al. (2023) demonstrated that hypodivergent subjects had significantly greater intermolar and intercanine widths compared to hyperdivergent subjects, while normodivergent individuals exhibited intermediate values [14]. This conclusion provides strong support to the current study, further cementing the belief that vertical facial type is a determining factor in dental arch dimensions.

Biomechanical analyses underscore these findings. Hypodivergent subjects typically have strong musculature and horizontal mandibular rotation, which are more conducive to transverse stability and greater arch dimensions. Hyperdivergent subjects have weaker musculature and vertical mandibular rotation, creating narrower arch forms, especially in the posterior segments. Normodivergent subjects are in the middle, having well-balanced arch measurements. This trend was evident in the current study.

The clinical significance of these findings is important. Dental arch expansion is more stable in hypodivergent patients because of advantageous skeletal and muscular support, while expansion in hyperdivergent patients is more prone to relapse. Retention of the original shape of the arch is hence important in orthodontic treatment planning. Choosing the archwire shapes should be based on consideration of the growth pattern of the patient in order to obtain long-term stable results.

In population relevance, the current work contributes to the body of literature by verifying analogous trends in the South Indian population. Even with ethnic variations affecting dental arch size, the correlation between growth pattern and arch width seems universal across populations, according to Chinese, American, and Indian sample-based studies [1,7,15].

A limitation of this study is that only dental arch widths were assessed without considering arch perimeter, depth, or shape, which may further influence occlusion and treatment outcomes. Additionally, the cross-sectional design does not allow evaluation of changes over time. Future longitudinal studies, including larger and more diverse populations, are recommended to validate these findings.

## Conclusions

The present study establishes a clear association between vertical growth patterns and dental arch widths. Hypodivergent individuals consistently exhibited the widest maxillary and mandibular arch dimensions, while hyperdivergent subjects demonstrated the narrowest. Normodivergent individuals maintained intermediate values, confirming a graded relationship across growth patterns.

These findings highlight the influence of skeletal morphology on arch form and its clinical importance in orthodontic planning. Selection of archwires and expansion strategies should therefore be individualized based on vertical facial type. Overall, considering arch dimensions can enhance treatment stability and minimize relapse.
